# Anti-Gluten Immune Response following *Toxoplasma gondii* Infection in Mice

**DOI:** 10.1371/journal.pone.0050991

**Published:** 2012-11-29

**Authors:** Emily G. Severance, Geetha Kannan, Kristin L. Gressitt, Jianchun Xiao, Armin Alaedini, Mikhail V. Pletnikov, Robert H. Yolken

**Affiliations:** 1 Stanley Division of Developmental Neurovirology, Department of Pediatrics, Johns Hopkins University School of Medicine, Baltimore, Maryland, United States of America; 2 Division of Neurobiology, Department of Psychiatry, Johns Hopkins University School of Medicine, Baltimore, Maryland, United States of America; 3 Department of Medicine, Columbia University Medical Center, New York, New York, United States of America; University of Oklahoma Health Sciences Center, United States of America

## Abstract

Gluten sensitivity may affect disease pathogenesis in a subset of individuals who have schizophrenia, bipolar disorder or autism. Exposure to *Toxoplasma gondii* is a known risk factor for the development of schizophrenia, presumably through a direct pathological effect of the parasite on brain and behavior. A co-association of antibodies to wheat gluten and to *T. gondii* in individuals with schizophrenia was recently uncovered, suggesting a coordinated gastrointestinal means by which *T. gondii* and dietary gluten might generate an immune response. Here, we evaluated the connection between these infectious- and food-based antigens in mouse models. BALB/c mice receiving a standard wheat-based rodent chow were infected with *T. gondii* via intraperitoneal, peroral and prenatal exposure methods. Significant increases in the levels of anti-gluten IgG were documented in all infected mice and in offspring from chronically infected dams compared to uninfected controls (repetitive measures ANOVAs, two-tailed t-tests, all p≤0.00001). Activation of the complement system accompanied this immune response (p≤0.002–0.00001). Perorally-infected females showed higher levels of anti-gluten IgG than males (p≤0.009) indicating that *T. gondii*-generated gastrointestinal infection led to a significant anti-gluten immune response in a sex-dependent manner. These findings support a gastrointestinal basis by which two risk factors for schizophrenia, *T. gondii* infection and sensitivity to dietary gluten, might be connected to produce the immune activation that is becoming an increasingly recognized pathology of psychiatric disorders.

## Introduction

Gluten proteins of wheat and related cereals have a pathogenic effect on the intestinal tract of individuals with the autoimmune disorder, celiac disease [1]. Increasingly, the exposure to gluten and to other food antigens such as bovine milk caseins is implicated in the pathogenesis of neuropsychiatric diseases, including autism, schizophrenia and bipolar disorder [2], [3], [4], [5], [6], [7], [8], [9], [10], [11], [12], [13], [14]. The digestion of wheat glutens and milk caseins has been proposed to result in the production of neuroactive exorphins that penetrate compromised gastrointestinal (GI) and blood brain barriers and directly bind to opioid receptors in the CNS [4], [8], [10], [15], [16], [17], [18]. We recently reported significant correlations of markers of GI inflammation with antibody levels to gluten and casein in individuals with schizophrenia [11]. Among these correlations, we found that antibodies to the targeted food antigens were associated with antibodies to the protozoan parasite, *Toxoplasma gondii*, in individuals with a recent onset of the disease.

*T. gondii* has been studied predominantly in the context of psychiatric disorders as a pathogen that might modify host behavior through a direct effect on the central nervous system [19], [20], [21], [22], [23], [24], [25], [26], [27], [28]. The association between food antigen exposure and the *T. gondii* parasite in individuals with schizophrenia presents a peripheral pathway via the GI tract by which this parasite may also contribute to psychiatric disease. In murine models, the oral ingestion of *T. gondii* can cause small intestinal immunopathology and is thus used experimentally to induce colitis and ileitis [29], [30], [31], [32], [33], [34]. In humans, increased titers of *T. gondii* antibodies have been found in individuals with inflammatory bowel disease and celiac disease [35]. In pregnant women, *T. gondii* infection rates were higher among those with celiac disease compared to those without a gluten sensitivity [36]. It is not possible to discern from these studies if GI pathologies preceded *T. gondii* infection or vice versa.

Here, we investigated the functional link between two previously unassociated schizophrenia risk factors, *T. gondii* exposure and immune reaction to gluten, in experimental mouse models. We also evaluated if adult and prenatal exposures to *T. gondii* might impact complement activation, a marker of systemic inflammation and antigen presence in the circulation [37], [38], [39], [40]. Results from these experiments demonstrate for the first time that intestinal *T. gondii* infection can lead to elevated antibody levels to dietary gluten. These findings lay the groundwork for future studies to explore how digested gluten peptides, independently or combined with *T. gondii* and other GI pathogens, might impact brain physiology and behavior.

## Materials and Methods

### Ethics Statement

Investigations were approved by the Johns Hopkins Animal Care and Use Committee Institutional Guidelines (permit #MO12M23). These studies were part of a larger ongoing project to evaluate genetic and environmental hypotheses of schizophrenia in mouse models. Utmost effort was utilized to prevent suffering and minimize the numbers of mice required for each experiment.

### Overview

**Figure 1:**
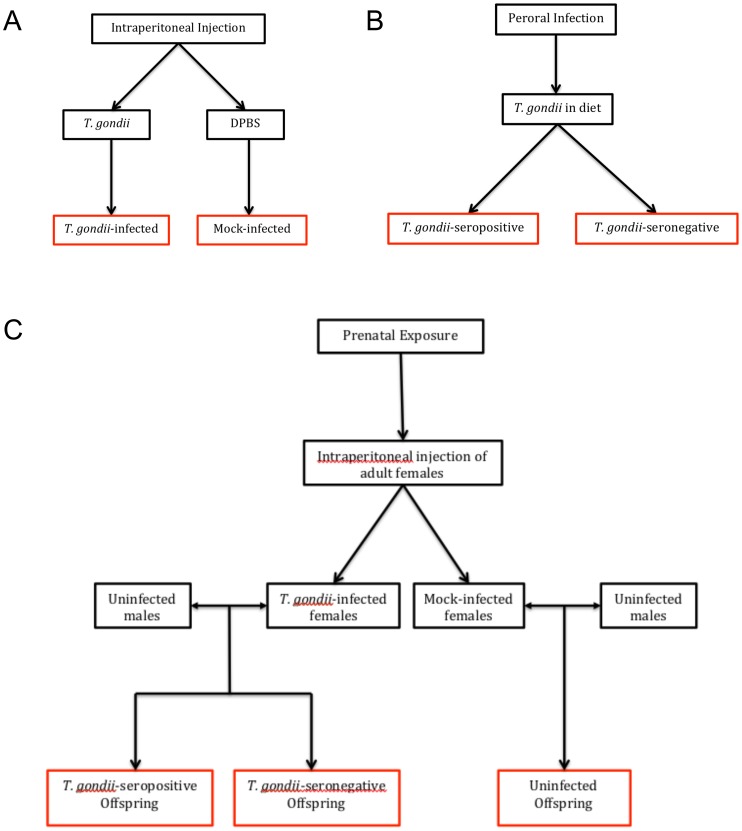
Overview of modes of *T. gondii* infection and comparison groups. Panel A: Adult mice received intraperitoneal injections as shown to generate a *T. gondii*-infected and a mock-infected group. Experimental groups are boxed in red. Panel B: Adult mice received diets containing *T. gondii*, which resulted in mice that were successfully infected with *T. gondii* (*T. gondii*-seropositive) and those that were exposed to but did not develop an infection (*T. gondii*-seronegative). Panel C: IP- and mock-infected females were mated to uninfected males to produce offspring that were (1) *T. gondii*-exposed and *T. gondii*-seropositive, (2) *T. gondii*-exposed and *T. gondii*-seronegative, or (3) uninfected.

Exposure of mice to *T. gondii* was achieved using three routes of infection: intraperitoneal (IP), peroral (PO), and prenatal. An overview of how the experimental groups were generated via each type of *T. gondii* infection is depicted in Figure 1.

### IP Infection

Twenty-four 8-week old Balb/C mice were injected into the peritoneal cavity with either 400 tachyzoites of the Type II *T. gondii* strain, Prugniaud (PRU; n = 6 male; n = 6 female), or with Dulbecco’s phosphate buffered saline (DPBS; mock-infected; n = 6 male; n = 6 female). Serum samples were collected from a tail clip at 0, 15 and 21 days post-infection (dpi). IgG levels to *T. gondii*, gluten and complement factor C1q were measured with enzyme-linked immunosorbent assays (ELISAs), as described below. Over the course of the study, four of the female IP *T. gondii*-infected mice died. These animals were not included in the analyses.

### PO Infection

A PO-infected cohort was generated with the goal of providing a more natural means of *T. gondii* infection and limiting to the extent possible any stress introduced by the procedure itself. Thus, in this PO model, we developed a diet containing infectious *T. gondii* mixed with a crushed rodent chow that contains as its primary protein ingredient ground wheat (2018SX Teklad Global 18% Protein Extruded Rodent Diet; Harlan Laboratories, Madison, WI, U.S.A.). Animals were then allowed to eat freely (i.e. they were not forced). This PO method of inoculation was expected to produce infection in only a subset of animals that received this diet.

To prepare infectious *T. gondii* material, 5- and 8-week old BALB/c and mixed background BALB/c crossed with C57BL6, F4 donor mice were injected IP with 400 tachyzoites of the *T. gondii* PRU strain. At five weeks post-infection, mice were sacrificed, their brains harvested, and serum obtained from a tail clip. Infectious brain homogenates were then mixed with crushed rodent chow.

Recipient mice were composed of two cohorts of BALB/c mice. Cohort 1 included twelve 12-week old mice (n = 6 male; n = 6 female); cohort 2 included ten 4-week old (n = 5 male; n = 5 female) and ten 6-week old mice (n = 5 male; n = 5 female). Recipient mice were food-deprived for 19 hours prior to infection. The food mixture of brain homogenate and crushed rodent chow was placed in the cages of all recipient mice, and mice were allowed to freely eat for three hours. Serum was collected at 4–5 weeks post-infection for cohort 1 and at 3 weeks post-infection for the cohort 2. These two cohorts were combined for the data analyses described below. Antibodies to *T. gondii*, gluten and C1q were measured via the ELISAs described below.

### Prenatal Infection

Female mice were injected IP with 400 tachyzoites of the *T. gondii* PRU strain. *T. gondii*-seropositive dams were mated with uninfected males. Eleven offspring were produced from this mating. The control group was composed of DPBS mock-infected dams mated with uninfected males. Four offspring resulted from this mating. Serum was taken from all offspring from a tail clipping at postnatal day 7. Antibodies to *T. gondii*, gluten and C1q were measured as described below.

### Laboratory ELISA Procedures

In serum samples, IgG antibodies to *T. gondii*, gluten and C1q were measured by ELISAs. Commercially available ELISA kits for measuring serological *T. gondii* IgG were purchased from IBL America (Minneapolis, MN, U.S.A.) and used as previously described with a 1∶50 serum dilution and an anti-mouse IgG secondary antibody [41]. IgG antibodies to wheat gluten and C1q were measured by ELISAs using previously described methods with some modification [11], [42]. Whole gluten was extracted from the wheat cultivar Cheyenne, also as previously described [14]. C1q was purchased from Sigma-Aldrich (St. Louis, MO, U.S.A.) Wells of 96-well microtiter plates were incubated with one hundred ng of protein in 50 µl carbonate buffer overnight at 4°C. The plates were incubated with serum samples diluted 1∶50 in PBST for 2 h at 37°C. Plates were washed and incubated with peroxidase-conjugated goat-anti-mouse IgG secondary antibody for 30 min at 37°C (Southern Biotech, Birmingham, AL, U.S.A.). 2,2′-azino-di-(3-ethylbenzthiazoline-6-sulfonate)(ABTS)/hydrogen peroxide solution (KPL Protein Research Products, Gaithersburg, MD, U.S.A.) was added for color development, and absorbance was measured at 405 nm, with a reference wavelength of 490 nm, in an automated microtiter plate reader (Molecular Devices, Menlo Park, CA, U.S.A.).

**Figure 2:**
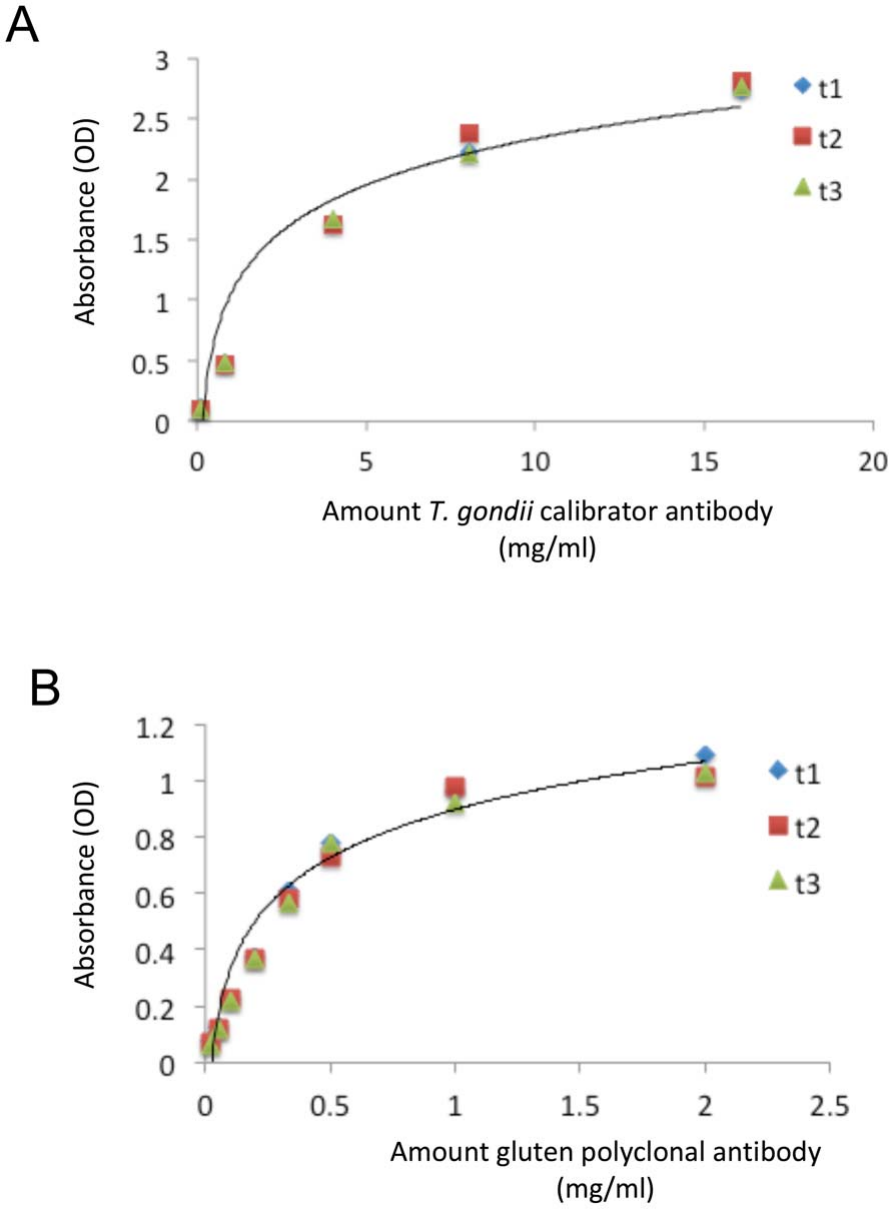
Standard curves from ELISAs for estimation of serological levels of anti-*T. gondii* and anti-gluten IgG. Panel A: The amount of *T. gondii* calibrator antibody is plotted according to absorbance. The standard curve shown is defined by the equation, y = 0.5342ln(x)+1.0478. The range of detection of *T. gondii* antibody for this assay is 100 ng-20 mg/ml. IP and PO inoculations produced *T. gondii* antibodies in excess of 20 mg/ml. Prenatal exposure to *T. gondii* resulted in IgG levels of around 1 mg/ml in offspring. Panel B: The amount of gluten antibody is plotted according to absorbance. The gluten antibody curve is defined by the equation, y = 0.235ln(x)+0.8572. The range of detection for this assay is 20 ng–2 ug/ml. IP, PO and prenatal exposures resulted in IgG levels of 50–90 ng/ml serological anti-gluten IgG. T1, T2, T3 refer to replicates of the assays run to construct these standard curves.

Using the above ELISA methods, standard curves of antibody levels (mg/ml) were generated for *T. gondii* using the calibrators available in the commercial kit and for gluten using a rabbit anti-gliadin polyclonal antibody (Sigma-Aldrich, St. Louis, MO, U.S.A.). The concentration of antibodies specific to *T. gondii* or to gluten was then calculated. For the *T. gondii* calibrators provided in the commercial kit, conversion of International Units to milligrams per milliliter was performed according to Humphrey and Batty [43]. Standard curves for determination of serum IgG estimates are found in Figure 2.

### Statistical Analyses

*T. gondii* seropositivity was established based on values from the ELISA kit control standards run with each assay. Anti-*T. gondii*, -gluten and -C1q IgG levels were compared between: (1) IP *T. gondii*-infected and IP mock-infected animals, (2) PO-infected *T. gondii*-seropositive and PO-infected *T. gondii*-seronegative animals, and (3) offspring of infected vs offspring of uninfected mothers. Repetitive measures ANOVAs were applied in the IP experiment to identify significant differences in antibody levels over time among groups according to sex and infection status. Statistical differences in mean levels of antibodies among groups and sex-specific subgroups in the PO and prenatal exposure cohorts were identified with two-tailed t-tests. P-values less than 0.05 were considered to be statistically significant. Statistical analyses were performed with STATA version 12 (STATA Corp LP, College Station, Texas, U.S.A.).

## Results

### Intraperitoneal (IP) Infection

**Figure 3:**
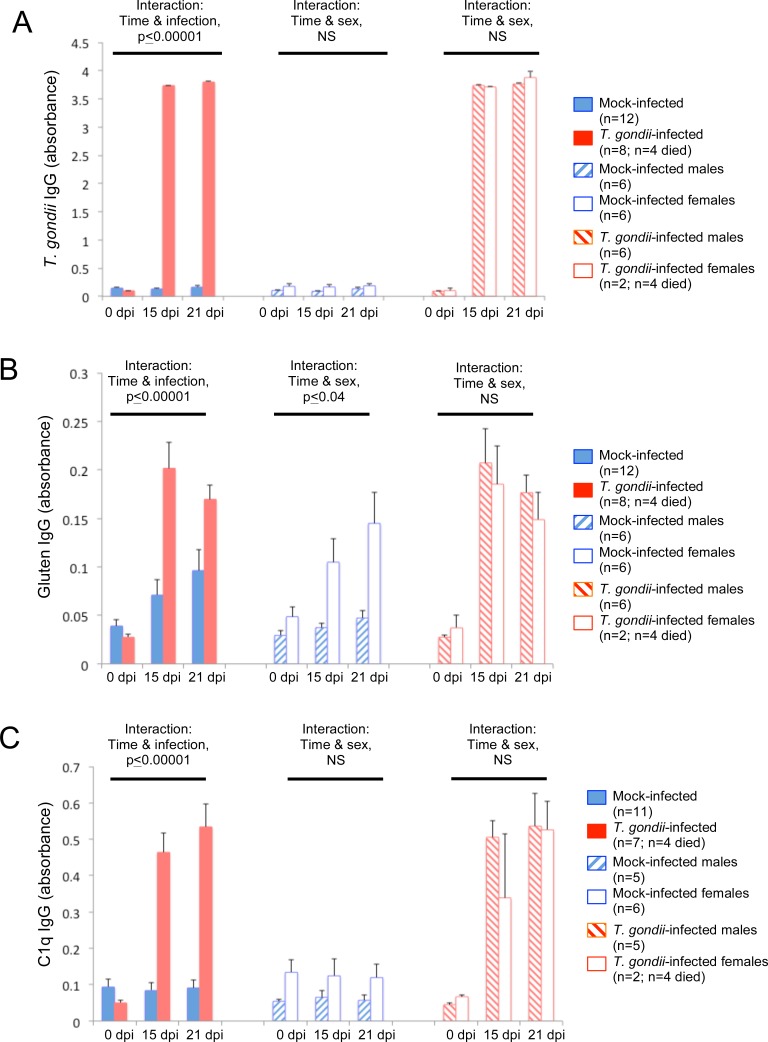
IgG antibody levels following intraperitoneal (IP) *T. gondii* inoculation. Panel A: Levels of anti-*T. gondii* IgG in mice infected IP with *T. gondii* (red) varied significantly over time compared to mock-infected control mice (blue). *T. gondii* antibody levels were not different between sexes (hashed bar – males; open bar – females). Panel B: Levels of anti-gluten IgG in mice infected IP with *T. gondii* varied significantly over time compared to mock-infected control mice. Between mock-infected male and female mice, anti-gluten IgG levels varied significantly over time. In IP *T. gondii*-infected mice, anti-gluten IgG levels over time were not significantly (ns) different between sexes. Panel C: Levels of anti-C1q IgG in IP-infected mice varied significantly over time compared to mock-infected control mice. Time, infection and sex refer to interaction variables in repetitive measures ANOVAs. Repetitive measures ANOVAs were used to generate listed p-values. Error bars indicate standard errors of the mean. NS refers to not significant. Dpi refers to days post-infection.

We first measured anti-*T. gondii* IgG levels to establish *T. gondii*-seropositive and *T. gondii*-seronegative groups. Following IP infection, highly statistically distinct *T. gondii-*seropositive and *T. gondii*-seronegative groups were generated. The IP route of infection resulted in *T. gondii* seropositivity in 100% of the animals that were inoculated with the parasite (n = 6 males; n = 6 females). Animals injected with a DPBS vehicle (mock-infected; n = 6 males; n = 6 females) were *T. gondii*-seronegative. Four of the six females from the IP *T. gondii* group died during the course of the experiment, and these animals were not included in the analyses. Anti-*T. gondii* IgG levels increased over time in *T. gondii*-inoculated animals compared to those that were mock-infected (Figure 3, panel A; repetitive measures ANOVA, between groups p≤0.00001; interaction of infection group and time since infection, p≤0.00001). There were no differences in *T. gondii* IgG between sexes in either the *T. gondii*-infected or mock-infected groups.

In IP-inoculated mice, anti-gluten IgG increased over time in animals infected with *T. gondii* (n = 8) compared to those that were mock-infected (n = 12; Figure 3, panel B; repetitive measures ANOVA, between groups p≤0.003; interaction of infection group and time since infection, p≤0.00001). When animal groups were broken down according to sex, the interaction of infection group and time remained significant for the males (n = 12; between infection groups, p≤0.0002; interaction p≤0.00001), but not females (n = 8; infection groups, p≤0.49; interaction p≤0.20). This difference between sexes in gluten antibody levels over time was attributable to the mock-infected group (Figure 3, panel B; sex p≤0.008; interaction sex and time, p≤0.04), whereas no difference in anti-gluten IgG levels over time was observed between sexes in the *T. gondii*-infected animals (Figure 3, panel B; sex p≤0.69; interaction sex and time, p≤0.72).

Complement C1q activation in IP-inoculated mice was significantly elevated compared to mock-infected mice and this response increased over time since infection (Figure 3, panel C; between infection groups p≤0.00001; interaction p≤0.00001; n = 7 *T. gondii*-infected, n = 11 mock-infected). Levels of C1q activation were not significantly different between sexes in the *T. gondii*- or mock-infected groups.

### Peroral (PO) Exposure

**Figure 4:**
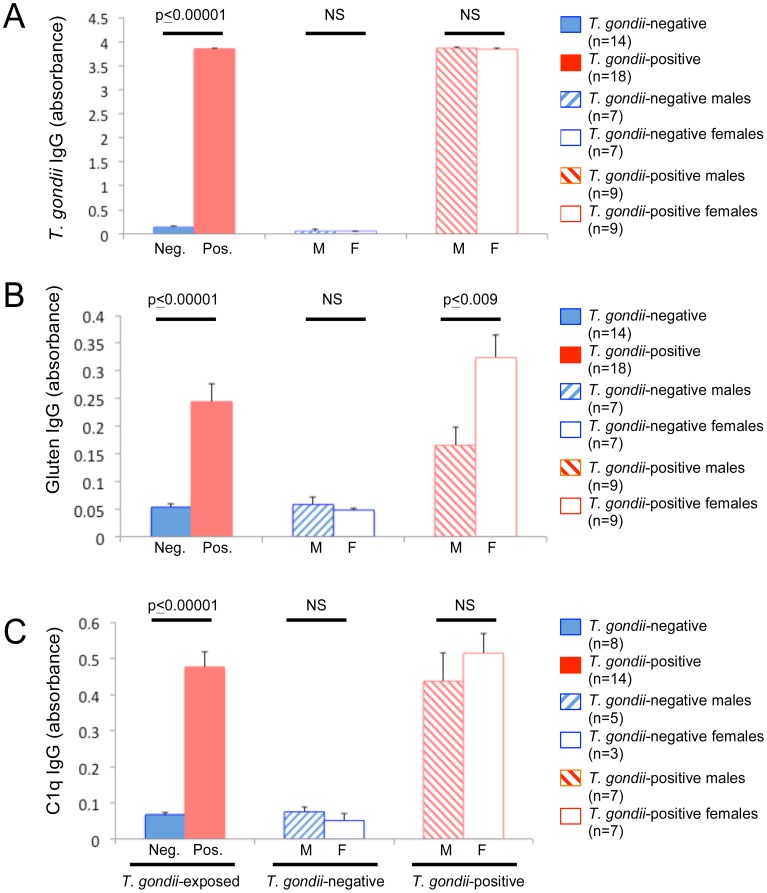
IgG antibody levels following peroral (PO) *T. gondii* exposure. Panel A: Levels of anti-*T. gondii* IgG were significantly greater in mice that were seropositive for *T. gondii* (*T. gondii*-positive) compared to those that were seronegative (*T. gondii*-negative) following ingestion of rodent chow containing *T. gondii*. Panel B: Levels of anti-gluten IgG were significantly greater in mice that were seropositive for *T. gondii* (*T. gondii*-positive) compared to those that were seronegative (*T. gondii*-negative) following ingestion of rodent chow containing *T. gondii*. No significant differences (NS) in anti-gluten IgG levels were found between sexes in the *T. gondii*-negative group. In animals that were *T. gondii*-positive, anti-gluten IgG levels were significantly higher in females compared to males. Panel C: C1q antibody levels were significantly greater in *T. gondii*-positive vs *T. gondii*-negative animals. No sex-specific differences were observed. Two-tailed t-test comparisons were used to generate listed p-values. Error bars indicate standard errors of the mean.

In animals exposed to *T. gondii* in their diet (PO), ten of 12 mice from the first cohort (12 week-old mice tested 4–5 wpi) and eight of 20 mice from the second cohort (4- and 6-week old mice tested 3 wpi) tested seropositive for *T. gondii*. The PO rate of infection using this method was thus 56.25%. Seropositive mice had significantly higher levels of anti-*T. gondii* IgG than those that were seronegative (Figure 4, panel A; two-tailed t-test, t = −1.7e^02^, p≤0.00001). There were no significant differences in anti-*T. gondii* IgG between sexes (seropositive, n = 9 male, n = 9 female; seronegative, n = 7 male, n = 7 female).

In animals orally exposed to *T. gondii*, mean levels of anti-gluten IgG were significantly greater in *T. gondii*-seropositive (n = 18) vs *T. gondii*-seronegative (n = 14) mice (Figure 4, panel B; two-tailed t-test, t = −5.16, p≤0.00001). In the *T. gondii*-seropositive group, anti-gluten IgG levels were significantly elevated in females compared to males (Figure 4, panel B; two-tailed t-test t = −3.00, p≤0.009). Gluten antibody levels were not significantly different between sexes in the mock-infected group (Figure 4, panel B; two-tailed t-test, t = 0.74, p≤0.48).

C1q antibody levels were significantly increased in *T. gondii*-seropositive mice (n = 14) compared to those that were seronegative (n = 8; Figure 4, panel C; two-tailed t-test t = −6.44, p≤0.00001). No sex-specific differences were detected.

### Prenatal Exposure

**Figure 5:**
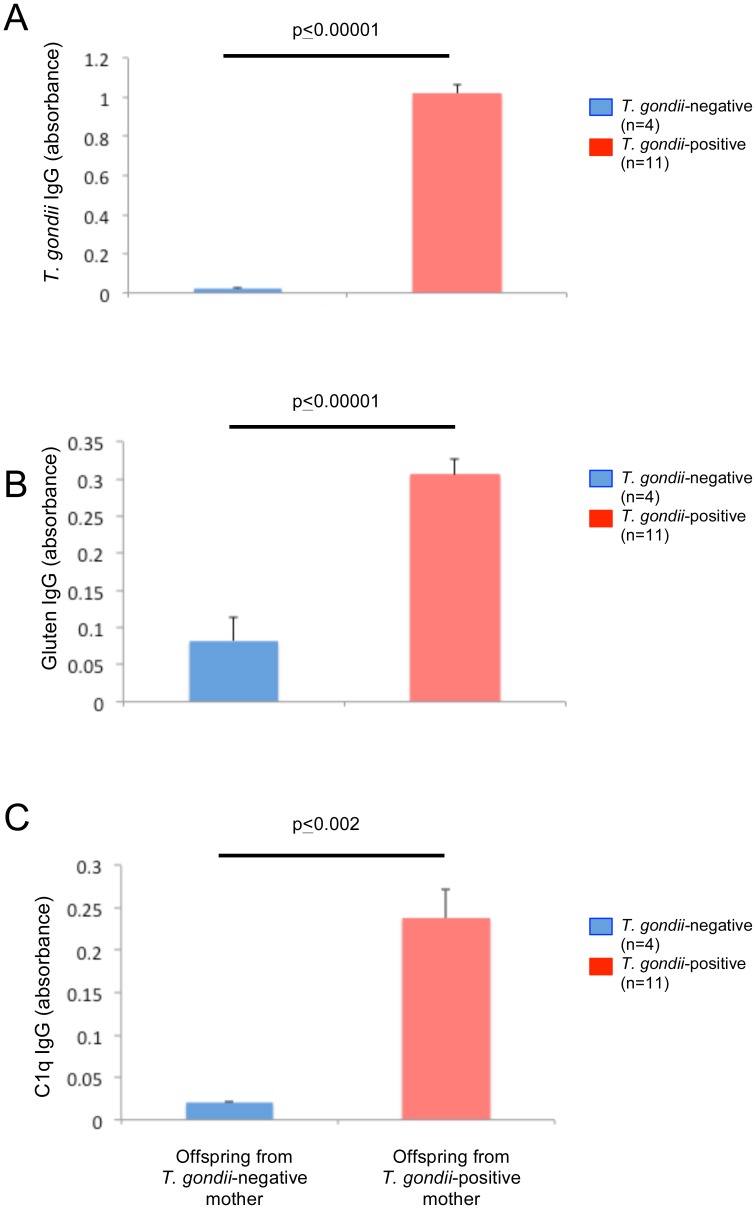
IgG antibody levels following prenatal exposure to *T. gondii*. Panel A: In 7-day old pups, anti-*T. gondii* IgG levels were significantly elevated in those whose mothers were seropositive for *T. gondii* compared to those whose mothers were uninfected. Panel B: 7-day old offspring exposed prenatally to *T. gondii* had significantly greater IgG to gluten than offspring from uninfected mothers. Panel C: 7-day old offspring of mothers who were seropositive for *T. gondii* had significantly stronger complement factor C1q activation than offspring from uninfected mothers. Two-tailed t-test comparisons were used to generate listed significant p-values. Error bars indicate standard errors of the mean.

Sexes were not determined for the 7-day old pups born in the prenatal cohort. Of the 11 mice born to the infected mothers, 100% were *T. gondii*-seropositive. The four mice born to uninfected mothers were *T. gondii*-seronegative. *T. gondii* IgG levels were significantly increased in the seropositive group (Figure 5, panel A; two-tailed t-test t = −8.91, p≤0.00001).

Mean gluten IgG levels of pups (n = 11) born to *T. gondii*-seropositive dams were significantly greater than levels of pups (n = 4) born to uninfected dams (Figure 5, panel B; two-tailed t-test, t = −7.73, p≤0.00001).

C1q antibodies were significantly increased in offspring of *T. gondii*-seropositive dams compared to offspring from seronegative dams (Figure 5, panel C; two-tailed t-test, t = −3.7, p≤0.002).

## Discussion

Our findings convincingly indicate a direct relationship between infection with *T. gondii* and the generation of antibodies to gluten. A standard laboratory rodent chow contained enough wheat to precipitate the anti-gluten immune response in infected animals. In particular, female mice infected perorally with *T. gondii* had a significantly stronger anti-gluten immune response compared to infected males or uninfected control animals. Female-specific effects were also found in IP-inoculated animals; however, this method of infection was associated with a high mortality rate and is itself a less natural means of infection by *T. gondii* than the peroral route. The anti-gluten immune response and activation of the complement system following prenatal exposure to *T. gondii* infection coincides with a critical time period of postnatal neuronal development. These results, therefore, offer important milestones for future experiments to evaluate how the combined exposure to infection and dietary antigens, both known risk factors for the development of schizophrenia, might contribute to neurodevelopmental hypotheses of psychiatric disease etiology.

Results from both the PO and IP portions of our study provide support that the female sex is more severely affected following *T. gondii* infection. Four out of the six females inoculated IP with *T. gondii* died, compared to100% viability in males infected using the same method. Indeed, others have shown that female mice are more susceptible to and more likely to die from *T. gondii* infection than males [44], [45]. Therefore, perhaps it is not surprising that PO *T. gondii* launched a stronger anti-gluten immune activation in female mice compared to similarly infected males. Even in the IP mock vehicle group of our study, females exhibited significant elevations of gluten antibodies compared to pre-injection levels; males in this group showed no significant anti-gluten immune activation. This sex bias following a seemingly innocuous laboratory procedure is in keeping with reports from several groups regarding differential immune responses by female vs. male BALB/c mice [46], [47], [48]. Drude and colleagues (2011) reported that BALB/c mice undergoing standard laboratory injections with non-toxic substances such as saline and cyclodextrin exhibited sex-specific differences in the stress response, which in turn resulted in stress-induced lymphocytopenia [46]. Our results confirm that routine laboratory methods such as the IP route of delivery are not benign, and that stress-induced immune activation might, in fact, be compounded by exposure to dietary food antigens. Gluten antibody elevations in the IP mock-infected group of female mice further illustrate that it is the integrity of the GI environment that contributes to this food antigen response, a condition that can be brought on by stress or as modeled here by *T. gondii* infection. It is likely that infection with other GI pathogens would also cause a level of GI dysfunction that would directly and/or indirectly through associated immune activation/inflammation, affect the intake of nutrients and/or production of dietary antigens. As such, the ability of other intestinally-active pathogens to generate diet-related antibodies should be examined in animal models and in humans.

It is well-documented that stress leads to increased intestinal permeability [49], [50], [51] which, in turn, brings to question the inflammatory state of the GI tract brought on by *T. gondii* infection. It is not known with certainty how *T. gondii* strains gain access to systemic circulation, but a para-cellular route affecting epithelial tight junction proteins is suspected [52], [53], [54]. Following PO infection of mice with *T. gondii*, parasites can be detected in the lamina propria and in Peyer’s patches of the small intestine by one hour post-infection, and parasites are detected in circulation by 48 hours post-infection [53]. PO *T. gondii* infection is used to generate ileitis and colonitis in mouse models, and therefore can promote an environment of inflammation that damages cells lining the GI tract [29], [34], [53], [55], [56]). Physical damage that compromises the GI barrier integrity in turn promotes systemic inflammation as intestinal bacteria are translocated into circulation [49]. In fact, Hand and colleagues recently established that PO infection with *T. gondii* directly causes bacterial translocation of resident commensals, loss of immune tolerance to resident microbiota and activation of microbiota-specific T cells and inflammatory effector cells [34].

If *T. gondii* crosses into circulation without damaging barrier integrity, then we must speculate on other means by which the anti-gluten immune activation originates. The para- vs trans-cellular travel of gluten peptides across the GI barrier is itself not well-understood, but evidence for gluten association with both routes exists [57], [58], [59]. Sapone et al demonstrated that celiac disease and gluten sensitivity were, in fact, two separate clinical conditions based on standard GI permeability tests, which showed that only celiac disease was associated with GI leakage [60]. It may be that gluten directs its own passage as evident by interactions of gluten peptides with the intestinal permeability mediator, zonulin and other tight junction proteins; however, gluten-associated transcytosis has also been documented [57], [58], [59], [61], [62], [63], [64], [65].

The anti-gluten immune activation following exposure to *T. gondii* has especially interesting implications for exploring possible neurodevelopmental mechanisms by which gluten sensitivity might be involved with diseases such as schizophrenia and autism. In these neurodevelopmental disorders, a food antigen pathology has long been suspected for a subset of individuals, yet direct evidence for prenatal gluten exposure and subsequent gluten involvement in the central nervous system has been elusive. In a large birth registry-based study in Sweden, elevated levels of maternal anti-gluten IgG were recently found to increase the risk of non-affective psychosis in offspring [66]. Our experiments demonstrate that exposure to *T. gondii* infection during fetal development results in an anti-gluten immune response in offspring. Furthermore, we document that the complement pathway is also activated during this time period. C1q was used here as a gauge for generalized systemic immune activation following infection. Of interest to psychiatric diseases, however, is the role of C1q in synaptic pruning during development [67], [68]. In the early postnatal days of mouse CNS development, cortical C1q mRNA expression is extensive compared to activity at postnatal day 30 [67], [68]. In our study, serological C1q was significantly elevated in postnatal day 7 offspring exposed to *T. gondii* compared to unexposed offspring. Future studies are planned to examine how such prenatal exposures might affect subsequent synaptic organization.

In summary, the models described in this paper provide appropriate experimental tools to examine the impacts of gluten peptides, *T. gondii* and associated immune activation on brain physiology. As we accumulate more information from analyses of clinical biomarkers, we can adapt these animal models to test the effects of dietary modifications and other types of infections on behavioral endpoints, the pharmacological outcomes of specific antipsychotics on immune system parameters, and the autoimmune responses triggered by *T. gondii* infection. Ultimately, we envision a translational system by which we can fully evaluate the interface of environmental perturbation and genetic predisposition as it relates to serious neurodevelopmental disorders such as schizophrenia, bipolar disorder and autism.
